# Cerebrospinal fluid dynamics along the optic nerve

**DOI:** 10.3389/fneur.2022.931523

**Published:** 2022-08-15

**Authors:** Jinqiao Sheng, Qi Li, Tingting Liu, Xiaofei Wang

**Affiliations:** ^1^Laboratory for Biomechanics and Mechanobiology of Ministry of Education, Beijing Advanced Innovation Center for Biomedical Engineering, School of Biological Science and Medical Engineering, Beihang University, Beijing, China; ^2^School of General Engineering, Beihang University, Beijing, China

**Keywords:** cerebrospinal fluid, subarachnoid space, optic nerve, glymphatic system, eye movement

## Abstract

The cerebrospinal fluid (CSF) plays an important role in delivering nutrients and eliminating the metabolic wastes of the central nervous system. An interrupted CSF flow could cause disorders of the brain and eyes such as Alzheimer's disease and glaucoma. This review provides an overview of the anatomy and flow pathways of the CSF system with an emphasis on the optic nerve. Imaging technologies used for visualizing the CSF dynamics and the anatomic structures associated with CSF circulation have been highlighted. Recent advances in the use of computational models to predict CSF flow patterns have been introduced. Open questions and potential mechanisms underlying CSF circulation at the optic nerves have also been discussed.

## Introduction

The cerebrospinal fluid (CSF) is primarily composed of water (99%) (1) and occupies the subarachnoid spaces, perivascular spaces, and ventricles of the central nervous system, including the spaces in the brain, spine, and optic nerve (ON) (2). The normal volume of CSF is 125–150 ml (3). It is responsible for transporting nutrients, neurotransmitters, and hormones to nerve cells and controlling the osmotic pressure between different chambers (4). The CSF plays a significant role in the clearance of metabolic wastes from the central nervous systems (3). Many brain disorders, such as Alzheimer's disease (AD), are caused by the interruption of the CSF bulk flow along its route of circulation (5). High CSF pressure in the cranium may partly contribute to hydrocephalus (6).

In the ON, interrupted CSF circulation, also referred to as compartment syndrome of the ON (7, 8), causes injury to the anterior visual pathway (9, 10). For instance, abnormal CSF circulation is shown to be associated with glaucoma, in which the retinal ganglion cells are progressively damaged (11). Although important, studies on the ON CSF flow are scanty. Morphologically, the ON subarachnoid space is a cul-de-sac structure. To permit bulk CSF exchange between the intracranial chamber and the ON subarachnoid space, CSF is required to flow out of the ON subarachnoid space from the same route that it flows in. Studies have shown that a possible CSF route is lymphatics in the dura of the ON sheath (12) and the ON CSF is able to flow into the ON parenchyma through a perivascular transport system. Unfortunately, detailed information on the CSF circulation patterns at the ON is still unknown.

In this review, the anatomy and circulation route of the intracranial and ON CSF system have been introduced with an emphasis on the ON. Interstitial fluid (ISF) in parenchyma can be rapidly transported by AQP4 at the apex of the astrocytes (13), and then transferred to the perivascular spaces to exchange with CSF. Potential links between ocular movements and CSF circulation at the ON have been discussed. The glymphatic system, which is the waste clearance system in the central nervous system that utilizes CSF, has been presented. Imaging technologies used to visualize the CSF dynamics and the anatomic structures associated with CSF circulation have been underscored. Recent advances in computational models for predicting CSF flow patterns have also been introduced.

### Anatomic structure of the CSF system

Various tissues and structures are involved in the generation, circulation, and absorption of CSF, including the choroid plexus, ependyma, cisterna magna, subarachnoid spaces, Virchow–Robin space (VRS), brain parenchyma, the hypothalamus in the intracranial space, dura, ON subarachnoid space, and ON parenchyma (14). In this section, the anatomy and key functions of the structures of the CSF system in the intracranial spaces and the ON have been introduced.

#### Cranial part of the CSF system

**Figure 1:**
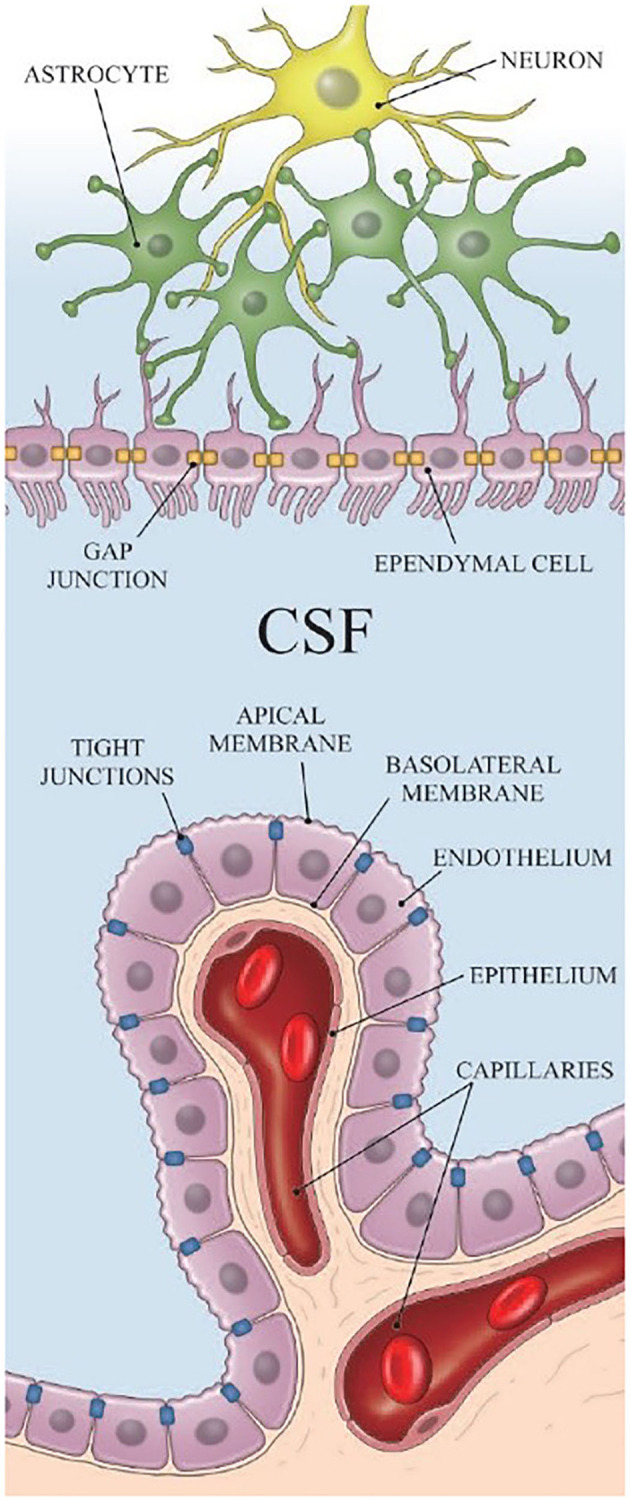
The structure of the ependyma [Reproduced from Nakada et al. (18) with permission from the publisher].

Most of the CSF is produced in the choroid plexus. The choroid plexus is located in the ventricular system, which is a set of interconnected cavities composed of two lateral ventricles, the third ventricle, the cerebral aqueduct, and the fourth ventricle. Capillaries, enfolded by fenestrated endothelial cells, form the core of the choroid plexus (15). The ependyma, which connects the cisterna magna to the choroid plexus, is found on the exterior of the choroid plexus (Figure 1) (16). CSF is produced in the capillaries, filling the ventricles and flowing out to the cisterna magna through slits between the ependyma. A major portion of the ependyma is covered by the brain–CSF barrier so that the CSF cannot pass through the ependyma directly. However, some parts of the ependyma are connected with the astrocytes of nerve cells in the cisterna magna to transfer substances between ISF and the CSF (17) (Figure 1).

CSF flowing from cisterna magna enters the subarachnoid space. The subarachnoid space is the space between the arachnoid mater and the pia mater, which is filled with CSF. It is occupied by delicate connective trabeculae and intercommunicating channels as well as branches of the arteries and veins of the brain (19).

**Figure 2:**
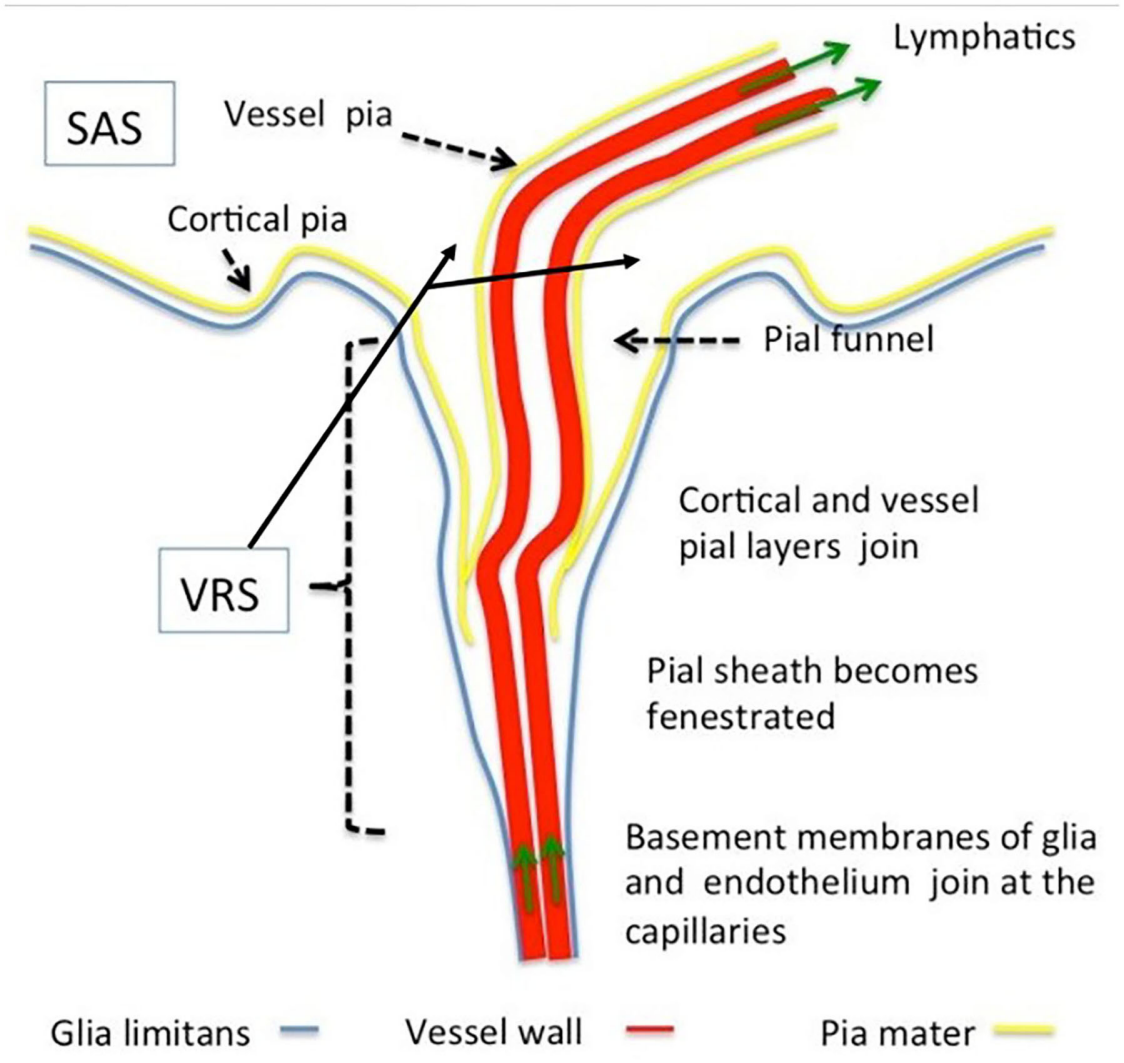
Morphology of Virchow–Robin and perivascular spaces. The Virchow–Robin space depicts the space surrounding vessels penetrating into the parenchyma with large turning angles [Reproduced from Brinker et al. (22) with permission from the publisher].

The VRS (Figure 2) are regarded as perivascular spaces containing perforating arteries extending into the brain parenchyma (20). Specifically, VRS refers to a histologically defined chamber, which surrounds the blood vessels penetrating into the brain tissues from the subarachnoid spaces. The structure of VRS is composed of endothelial, pial, and glial cell layers, each of them delineated by distinct basement membranes (21). The glial membrane, which covers the brain parenchyma, forms the walls of the VRS (22). VRS has now been described as part of the so-called glymphatic system.

There are three layers of meninges covering the central nervous system. The pia mater directly enfolds the parenchyma. Dura mater is the exterior most layer of the brain meninges. Between the dura and the pia is the arachnoid layer. Studies have shown that a network of lymphatic vessels exists in the dura, which is capable of draining CSF out of the brain (23, 24). However, this route of CSF drainage is controversial as there is a tight arachnoid barrier layer between CSF and the dura (25). More research is needed to determine the mechanism and importance of the dural lymphatic pathway for CSF outflow.

The last portion of the cranial part is the hypothalamus, one of the boundaries of the third ventricle. The hypothalamus links the nervous system to the endocrine system (26), transferring hormones to and from the CSF. For instance, hypothalamic 2-deoxy-2-(^18^F) fluoro-D-glucose ([^18^F]FDG) secreted by the hypothalamus takes part in the regulation of metabolic processes and sleep processes (27) *via* CSF circulation.

#### The structures of the ON

The length of the ON varies widely and is approximately 42–47 mm (28). Based on its structural characteristics, the ON can be divided into four portions: intraocular portion, intraorbital portion, intracanalicular portion, and intracranial portion (14). The ON enters the intracranial space through the optic canal. The subarachnoid space of the ON is partially connected to the intracranial subarachnoid space at the optic canal and ends at the ON–globe junction.

**Figure 3:**
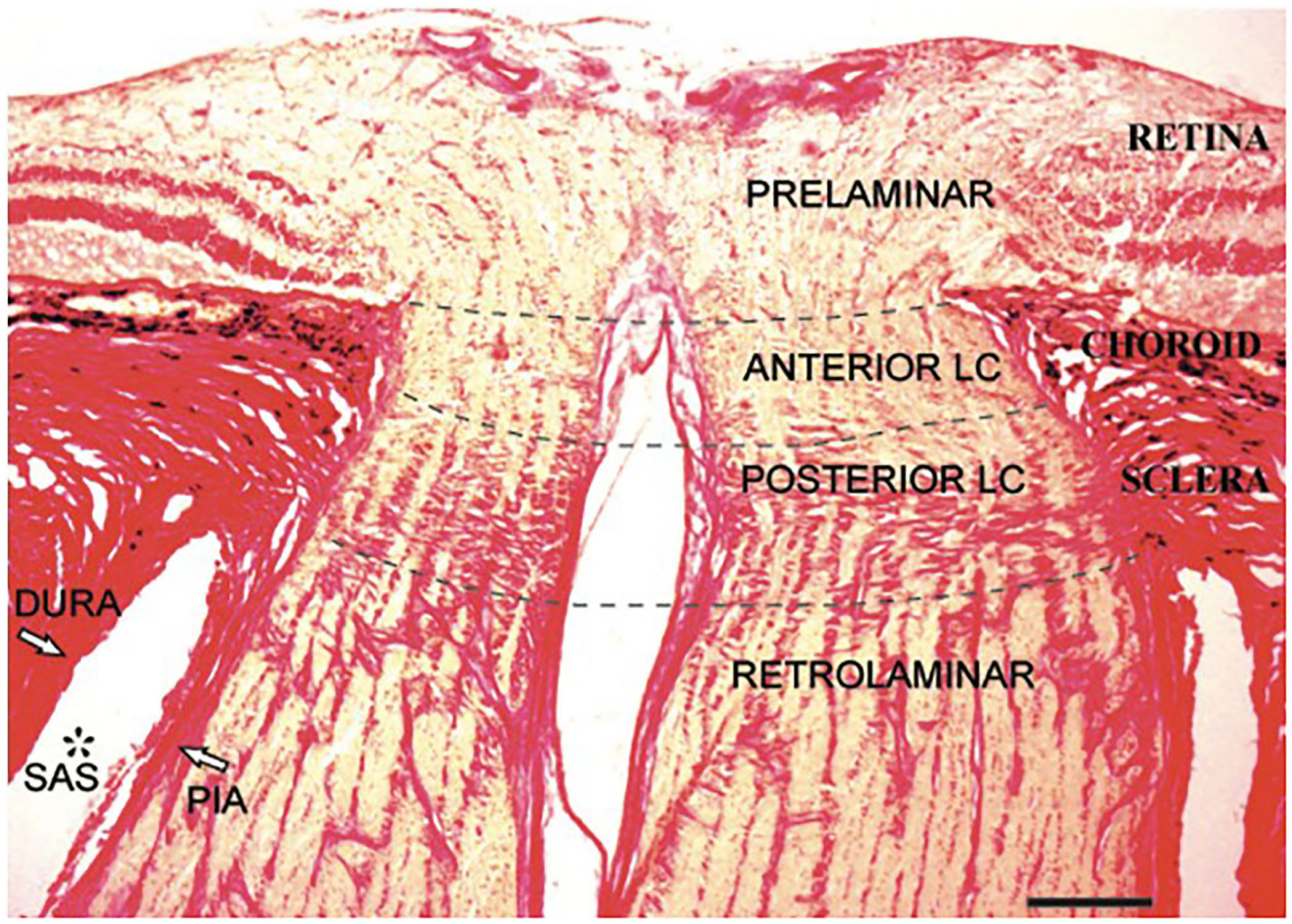
Histological section of the human intraocular portion in Van Gieson stain. LC: lamina cribrosa [Reproduced from Kang et al. (30) with permission from the publisher].

The intraocular portion (Figure 3) is the distal end of the ON, where the ON fibers converge and exit the eyeball. The intraocular portion is approximately 1 mm long and 1.5 mm (1.18–1.75 mm) in diameter. The lamina cribrosa within the intraocular portion is a connective tissue that bears the mechanical loads acting on this region together with the sclera. It is usually concave posteriorly. Retinal ganglion cell (RGC) axons exit the eyeball through the pores of the lamina cribrosa in bundles. Abnormal CSF circulation at this site can result in RGC apoptosis (29). The lamina cribrosa and peripapillary sclera separate the intraocular chamber from the subarachnoid space of the ON, which can result in a pressure gradient between the intraocular fluid and CSF in the ON.

The intraorbital portion of the ON is enveloped in meninges consisting of three layers: dura, arachnoid, and pia mater. The ON in this portion is slightly S-shaped (31). The width of the subarachnoid space in this portion becomes narrower from the retrobulbar portion to the intracanalicular portion. The diameter of this portion can be used to estimate the CSF pressure (32), which is usually measured using computed tomography, magnetic resonance imaging, and ultrasound imaging. The diameter of this portion is in the range of 5.17 ± 1.34–3.55 ± 0.82 mm (33).

**Figure 4:**
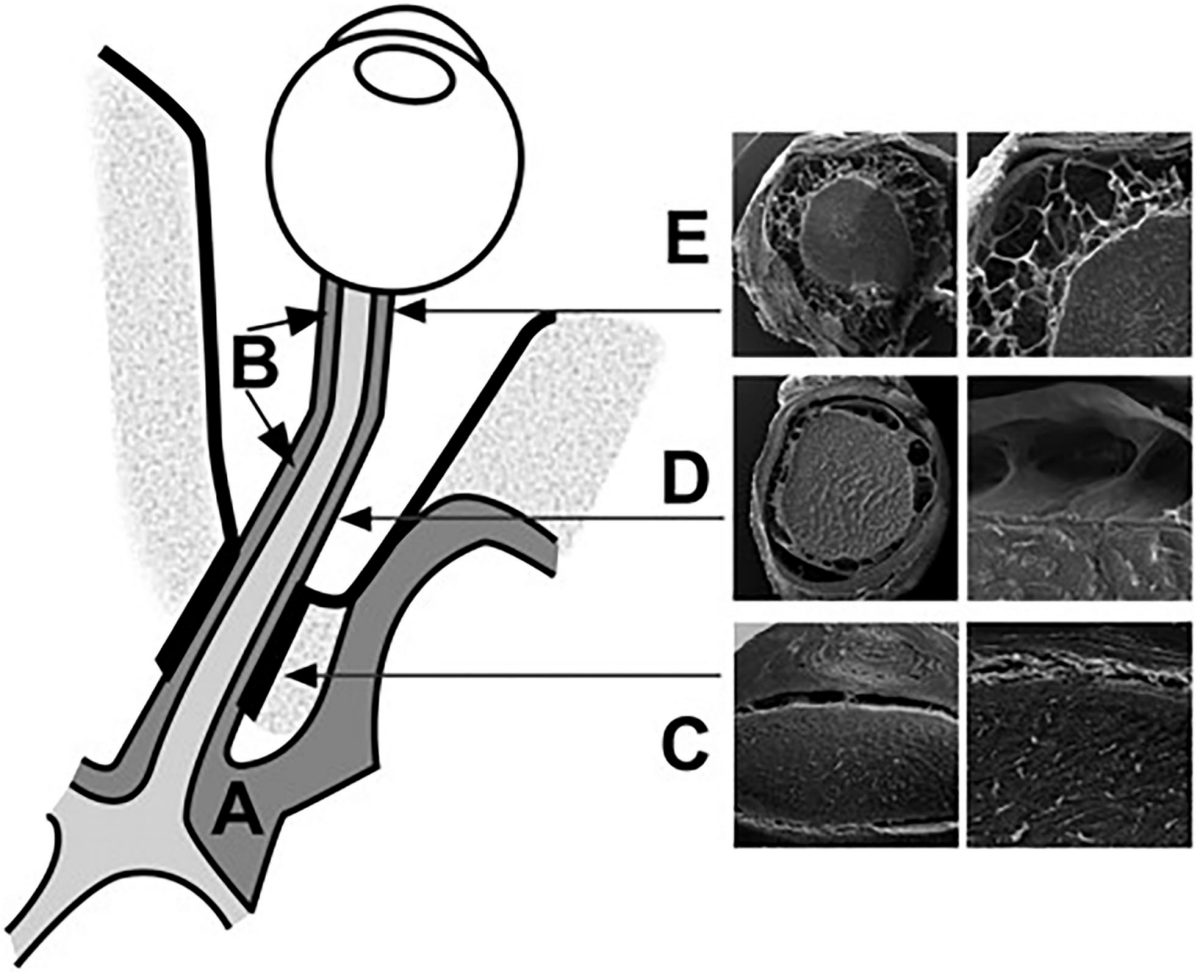
Schematic representation of the CSF spaces surrounding the optic chiasm **(A)** and the ON **(B)**. The subarachnoid space at the intracanalicular region is the narrowest **(C)**. The intraorbital segment of the SAS is characterized by broad septae **(D)**. The retrobulbar segment is characterized by small trabeculae **(E)**. The bulbar segment from the intracranial SAS to the orbital SAS [Reproduced from Killer et al. (35) with permission from the publisher].

The subarachnoid space in the intracanalicular portion connects with that in the intracranial portion. It is separated by one or two large pillars of approximately 0.5 mm containing one or two blood vessels (Figure 4). The subarachnoid space of the ON in this portion is the narrowest among the four portions of the ON. The arachnoid mater merges with the pia mater in the optic canal, suggesting a reduced free circulation from the subarachnoid spaces to the intracranial cavity and the ON subarachnoid layer in the human (34).

## CSF flow routes

### CSF flow routes in the brain

CSF is primarily produced in the choroid plexus and most of it flows to the third and fourth ventricles (36). Besides the choroid plexus, one study based on perfusion experiments suggested that nearly 30% of the total CSF production may arise from the ependyma, which is composed of the cuboidal epithelium (22). The ISF secreted at the capillary–astrocyte complex of the blood–brain barrier (BBB) is mixed with extracellular fluid and integrated with CSF (37). The capillary–astrocyte complex of the BBB is an active producer of CSF from the brain ISF (22).

The precise absorption sites of CSF are still not known. The entire parenchyma was once considered the absorption site of CSF. Recent studies by Nedergaard's group showed the glymphatic system, which is located deep within the brain parenchyma, might be the eventual pathway for CSF outflow (38). However, with advances in imaging technology, the arachnoid villi in the third ventricles have also been suggested to be the absorption site for CSF. Several studies have shown that CSF passes through the villi into sagittal sinuses (22).

In general, CSF flows from epithelial cells in the choroid plexus, through the VRS, hypothalamus, spinal cords, and eventually to the arachnoid villi of the third ventricle (39).

### CSF flow routes in the ONs

**Figure 5:**
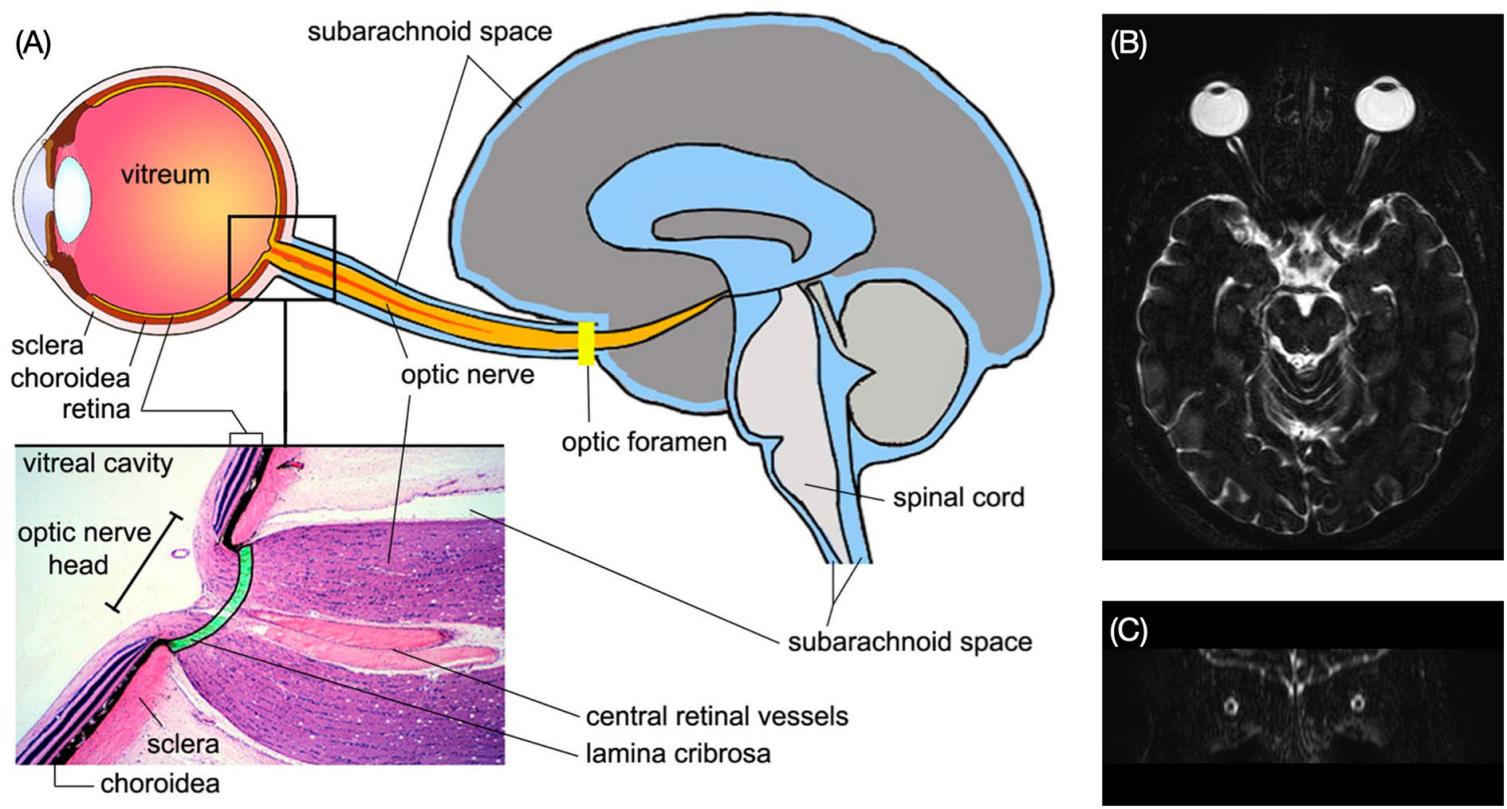
**(A)** Structure of the subarachnoid spaces of the ON and the brain. Enlarged view shows the structure of the ON head [Reproduced from Wostyn et al. (29) with permission]. **(B)** Axial MRI image of the brain shows the ON subarachnoid space in bright signal. **(C)** The ON subarachnoid space in coronal plane (Credit: Quan V. Hoang).

The cranial subarachnoid space is connected to the ON subarachnoid space (Figure 5) (40). Due to the volume gradient between the intracranial space and the subarachnoid space of the ON, the flow is expected to be unidirectional from intracranial spaces toward the ON in most scenarios (41). However, CSF is also able to flow back to the intracranial space if the pressure of the ON subarachnoid space is higher than that of the intracranial space. It is worth noting that CSF flowing in the ON subarachnoid space is a blind pouch, i.e., CSF needs to flow out from the same route it flows in. According to Golzan et al. (42), the CSF flow pulsation corresponds to the arterial flow in the ON, whereas the CSF reflection flow, referring to the CSF flowing back from the lamina cribrosa, is concurrent with the venous flow. This phenomenon suggests that the blood flow pulsation is the driving force behind CSF flow in the ON. However, in patients with normal tension glaucoma, the CSF flow in the ON subarachnoid space is considerably less than that in healthy subjects (43) but their blood flow is still normal, suggesting that blood flow is not the only driving force in CSF flow. Moreover, Morgan et al. (44) found that the retrolaminar tissue pressure is not relative to intracranial CSF pressure all over the time, i.e., CSF flow in the ON does not correlate to the arterial pulse in the ON. With this finding, CSF flow in the ON likely has its unique driving force for CSF flow, which is independent of intracranial blood flow.

It is speculated that ON dural sheath is involved in CSF transport through the lymphatic vessels in the dura (12). However, similar to the brain, the ON dura is covered by the arachnoid mater composed of cells with tight junctions, which is able to isolate the CSF of the ON subarachnoid space from the dura mater (45). It has been demonstrated in mice that a CSF tracer was not found near the ON (46). Therefore, the CSF circulation at the ON is still a matter of debate.

Inside the ON parenchyma, a perivascular transport system exists to allow CSF communications between the ON subarachnoid space and the ON parenchyma (47). Cells, such as astrocytes, with aquaporin-4 (AQP4; water channel proteins), can actively transport CSF out of the ONs (39) through AQP4 water channels into the ISF, which can be considered a part of the glymphatic system in the ON.

#### Eye movements impact the CSF flux in the ON

Human eyes exhibit frequent movements in daily activities with approximately 170,000 saccades per day (48). The angle of eye movement is approximately up to 44.9 ± 7.2° in adduction, 44.2 ± 6.8° in abduction, 27.9 ± 7.6° in elevation, and 47.1 ± 8.0° in depression (49).

Interactions between the eye globe and the ON (50–53) during eye movements could squeeze the ON and generate a higher pressure in the ON subarachnoid space to assist in the outflow of CSF. As a result of the unidirectional pathway of the ON CSF stated above, the pressure gradients that are generated are indispensable for the exchange of CSF between the subarachnoid spaces of the ON and the brain.

Of note, the eye rotates frequently during rapid eye movement (REM) sleep, which is a sleep stage, despite the eyes being closed. This state persists for 10–30 min at a time (54). Throughout the night, there may be 2–3 h of REM sleep in total (55). The angle of eye movements can reach 55° in REM sleep (49) which could deform the subarachnoid space of the ON and affect CSF circulation.

## Glymphatic system

Fluid in the lymphatic systems (56) plays a significant role in the removal of potentially toxic metabolic by-products. However, no conventional lymphatic systems are found in the parenchyma of the central nervous system.

Microscopically, the metabolic waste of brain tissues is cleared by a brain-wide perivascular pathway along which there is an exchange of CSF and ISF through AQP water channels (57), which is based on Nedergaard's findings (38). This system is referred to as the “glymphatic” system for its dependence on glial water channels and clearance functions, which are similar to those of the peripheral lymphatic system (38).

The glymphatic system provides novel insights into the pathology of AD. The deposition of β-amyloid plaques (11) in AD results in disordered Ca+ transferal in AQP4 water channels, blocking the transport of CSF and metabolic failure in the brain. The glymphatic system may be a promising turning point for elucidating and curing AD with further studies focusing on AD and the glymphatic system.

The ON is part of the central nervous system. Therefore, the glymphatic system should also exist in the ON to assist in the clearance of metabolic wastes. The ON glymphatic system can assist in understanding the development of glaucoma (11). In Wang's research, the transfer of HiLyte-594–tagged human β-amyloid (hAβ) tracer through CSF can also be found around RGCs (11). In the rodent brain model of AD, β-amyloid plaques can be observed during RGC apoptosis, which is a common symptom of glaucoma. All these phenomena support the pathological similarity of AD and glaucoma, implying a glymphatic system disorder in ON.

A higher trans-lamina cribrosa pressure difference (TLCPD) will cause deformation of the intraocular portion of the ON and distort the pathways of the ON glymphatic system, which could hinder the metabolic clearance mechanism and result in glaucoma (11). The intraocular portion is compressed by two pressures, the intraocular pressure (IOP) in the eye globe and the CSF pressure in the ON subarachnoid space (ON-CSFP). The lamina cribrosa and peripapillary scleral flange separate these two pressurized chambers (58). TLCPD is an important parameter that measures the pressure difference between the IOP and CSF pressure. A higher TLCPD induced by either a higher IOP or a lower CSF pressure is believed to be related to glaucoma development. The intracranial pressure (ICP) of normal subjects is measured at 7–15 mmHg in supine adults (59). As discussed above, CSF enters the ON subarachnoid space through the intracanalicular portion of the ON within the optic canal. Therefore, theoretically, ON-CSFP should be equal to the intracranial pressure (ICP). However, limited experimental measurements showed that in normal dogs, ICP is linearly correlated to ON-CSFP with ON-CSFP at 60% of the ICP (60). However, when ICP was reduced by 30%, this linear relationship between ON-CSFP and ICP disappears. One explanation for the differences in ON-CSFP and ICP is that the connection between these two chambers is reduced due to the narrow subarachnoid space at the intracanalicular ON. Reduced connections between these two chambers can induce an impeded CSF flow. Humans might likely have a different relationship but currently, there were no experimental data. Further studies are needed to elucidate the exact relationship between ICP and ON-CSFP in humans. Elevated ICP could cause the enlargement of ON subarachnoid space and papilledema. Therefore, the elevation of ICP can be observed qualitatively by the optic nerve sheath diameter through MRI, CT, and ultrasound imaging (61, 62).

Owing to the limitations of currently used imaging technologies and anatomical findings, whether the glymphatic system has an impact on those diseases is still unknown. Further research is thus required.

## Imaging techniques to visualize the CSF circulation

Various imaging technologies with corresponding indicator elements or substances can be utilized to visualize the pathways of CSF to understand its properties and functions. These techniques include magnetic resonance imaging (MRI), two-photon imaging, computer-assisted cisternography (CT cisternography), and scanning electron microscopy (SEM). Moreover, phase contrast techniques are also widely used in the CSF study (63).

### Magnetic resonance imaging (MRI)

**Figure 6:**
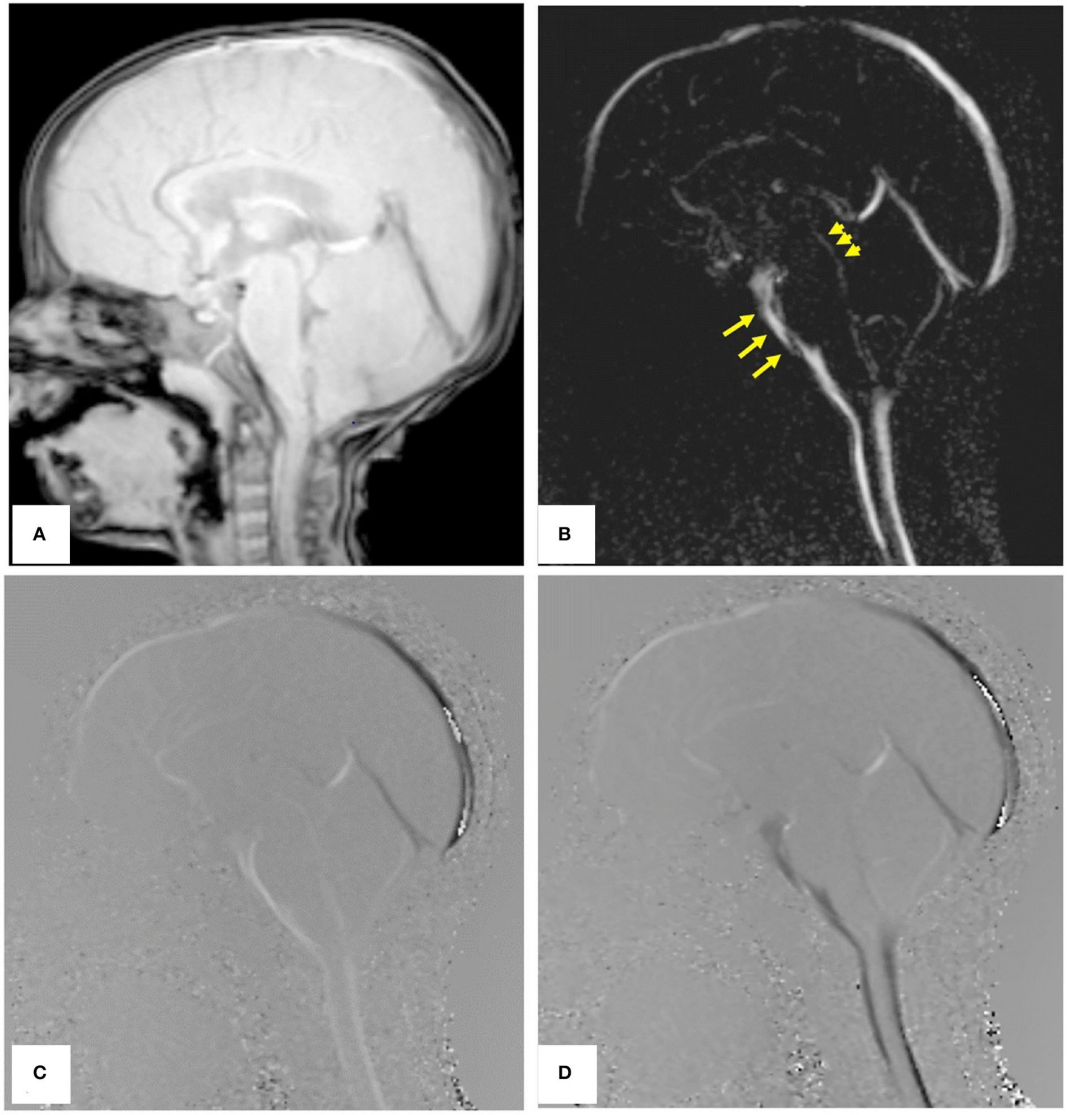
PCMRI image of a subject in the sagittal plane. **(A)** Re-phased image to show the background. **(B)** Magnitude image in which the flow is bright. **(C,D)** Are phase images showing the oscillation of CSF flow with bright and black color throughout the cardiac cycle [Reproduced from Mohammad et al. (67) with permission].

Phase-contrast MRI (PCMRI) is a commonly used method in studying CSF dynamics (64). PCMRI is an MRI technique that can be used to visualize the CSF movement, which was pioneered in the 1980s (65, 66). Figure 6 (67) shows PCMRI images in the sagittal plane to demonstrate the CSF flow. Later in the 2010s, a three-directional phase contrast MRI (4D Flow) has been developed to measure CSF flow both in in-plane directions and through-plane directions with higher spatial and temporal resolutions compared to conventional PCMRI (68). Recently, real-time PCMRI was developed, which allows a flow sampling rate of higher than 10 Hz (69). This frequency enables the measurements of CSF flows driven by heartbeat, respiration, and coughing in real-time. PCMRI was also employed in the study of CSF flow between the intracranial space and the ON. For instance, in Boy et al's. research (43) with PCMRI, patients with normal tension glaucoma were shown to have a lower flow-range ratio, demonstrating a possible role of impaired CSF dynamics in normal tension glaucoma.

Besides PCMRI, time-spatial labeling inversion pulse (Time-SLIP) is another technique that can visualize the CSF movement (70). It is useful for visualizing several seconds of the CSF flow pattern, providing CSF dynamics information even in the slow-flowing region (71). Clinically, it has the potential to determine whether CSF can flow from one compartment to another, which makes it a useful tool in the study of the CSF bulk exchange between intracranial space and the ON subarachnoid space.

Echo planar imaging (EPI) is another technique frequently used in MRI. EPI is capable of acquiring an image in milliseconds, allowing it to image dynamic physiologic processes such as CSF movement. In 1990, Stehling and associates showed that EPI with a snap-shot imaging time of 128 ms allows detailed demonstration of transient intraventricular CSF flow patterns (72). It also plays a crucial role in functional MRI (fMRI). Recently, EPI-based fMRI was frequently used to measure CSF pulsation during sleep and awake (73–75). fMRI differs from conventional MRI, which detects the energy signal of the resonant spinning hydrogen nucleus, with fMRI makes use of blood oxygen level-dependent (BOLD) contrast (hemoglobin) (76). Metabolic activities cause changes in the relative levels of oxyhemoglobin and deoxyhemoglobin in oxygenated or deoxygenated blood. Fast acquisition paradigms enable fMRI to detect fluid inflow. Specifically, fresh fluid arriving at the edge of the imaging region has high signal intensity because it has not yet experienced radiofrequency pulses. Therefore, by placing the imaging volume boundary at the region of interest (e.g., the fourth ventricle), fMRI can measure the dynamics of CSF flow (75). This imaging technology is widely used to detect neuronal metabolic functions in human brains. For instance, in a recent study by Fultz et al. (75), fMRI was used to examine the CSF flowing rate signals in human brains while sleeping, showing that CSF refreshes periodically. It also showed that in non-REM sleep, the hemodynamics and CSF metabolic dynamics are coupled. Moreover, the fMRI is used to test the difference between CSF flow signals in motor tasks and visual tasks in Kim et al's. research (74). The temporal resolution of fMRI is low compared to other conventional methods (77, 78). However, it allows the detection of hemodynamic oscillations and CSF movement simultaneously, thus allowing the study of the coupling between hemodynamics and CSF dynamics.

Because a contrast agent is not necessary for MRI, potential artifacts induced by contrast agent injection, such as the increase in the CSF volume, can be avoided. However, in a complex CSF flow pattern, because anterograde and retrograde flows mix, they may not be detectable with fMRI as a result of spatial overlap.

### Two-photon imaging

**Figure 7:**
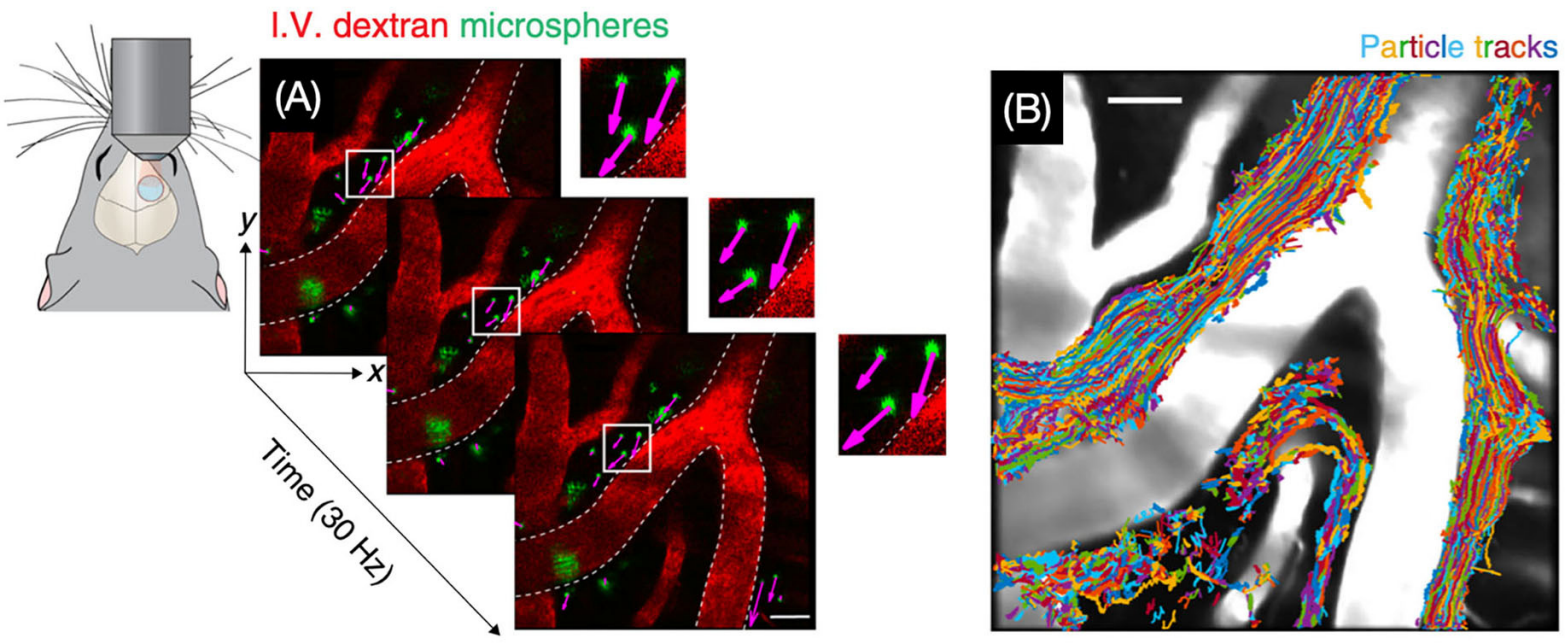
Visualization of CSF bulk flow in the perivascular space through two-photon microscopy. **(A)** Images for particle tracking velocimetry were acquired at 30 Hz. **(B)** Trajectories of tracked microspheres were superimposed on the image, showing that particles are transported within perivascular spaces [Reproduced from Mestre et al. (80) under a creative commons license].

Two-photon imaging is a type of fluorescence imaging with a limited imaging depth of approximately 300 μm (79). Because the penetration depth of two-photon imaging is confined to the skull, imaging of the CSF flow in the intracranial perivascular spaces usually requires invasive methods such as drilling a hole in the skull (Figure 7) (80). Iliff et al. (57), for instance, made use of *in vivo* two-photon imaging to detect the perivascular CSF pathway through the brain parenchyma. In this study, it is found that CSF enters the parenchyma along perivascular spaces, which surround the penetrating arteries and the ISF is cleared along perivenous pathways.

Ultimate three-dimensional imaging of solvent-cleared organs technique is usually coupled with two-photon imaging (11). This technology makes the tissues look transparent. As a result, the imaging depth can be deeper than that *in vivo*. The study conducted by Wang used this technique and found that the ocular β-amyloid clearance was affected by glial water channel AQP4 (11). Two-photon imaging is also frequently used in the observation of *ex vivo* brain slices to study the tracer distributions in the brain (81).

### CT cisternography

CT cisternography is an imaging technique used to visualize CSF through a contrast agent. Killer et al. have used this technique to determine the CSF dynamics between the intracranial space and the ON subarachnoid space in subjects with papilledema. CT cisternography showed a progressively reduced influx of CSF from intracranial CSF space to ON subarachnoid space (32). In another study, CT cisternography was used to measure the CSF dynamics along the ON in patients with idiopathic intracranial hypertension and normal subjects (82). Results showed that in patients with idiopathic intracranial hypertension and papilledema, CSF flow at the orbital ON is impaired.

Fleischman et al. (83) developed a procedure to image orbital CSF dynamics with CT imaging. In their procedure, a region of interest (ROI) is placed in the optic canal and at the end of the contrast front. When the superior and inferior parts of the nerve sheath are highlighted by the contrast front, a separate ROI is placed at the initiation of the nerve from the optic canal and at its termination in the inferior or superior location, respectively. A separate scan is used to determine the distance between the optic canal and the termination of the nerve. In Fleischman's research (83), the CSF flow velocity is the slowest in anesthesia. From the phenomenon, they surmized that eye movement, which can be restricted by anesthesia, maybe the accelerator of CSF flow (83).

Unlike MRI, contrast agents are essential to observe the aqueous pathways; otherwise, the bones and muscles with high tissue density will scatter the imaging contrast. Moreover, the resolution of CT is lower than that of two-photon imaging technology (84). Above all, the radiation of CT will be detrimental to experimental subjects.

### SEM

SEM is a powerful tool to visualize the microstructure of CSF pathways, which enables the understanding of potential patterns of CSF circulation in a tiny structure, such as in the ON, in terms of histological properties. In Killer's research, SEM was used to compare the microscale structure of different parts of the ON such as the intraorbital, retrobulbar, and bulbar segments (35). As a result, he found that the subarachnoid space of the ON is most narrow in the canalicular region with the complex structure of trabeculae and septae. This structure will make the exchange of ON CSF and intracranial CSF restricted. Nevertheless, SEM can only be performed ex vivo, thereby making it impossible to use it for real-time observations.

## Computational models to predict CSF flow patterns

Computational models are useful tools to simulate the biological system, especially in scenarios that are difficult to investigate using experiments (85). Focusing on the variation trends or microevolution, computational models are now widely used in explaining complicated mechanisms and deducing the effects of the CSF flow in structures.

Computational fluid dynamics (CFD) is a branch of fluid mechanics that utilizes equations governing a fluid motion to simulate complex fluid flows. Generally, four steps are required in a CFD simulation that are as follows: 1) constructing the geometry of the problem, usually through computer-aided design or reconstruction from medical images. 2) dividing the volume of fluid and the boundaries into discrete cells (elements). 3) defining the physical modeling such as the equations of fluid motion. 4) defining the boundary conditions (86). All computational models need to be validated against experimental results to provide reliable predictions.

Computational models are versatile in terms of predicting the CSF properties in perivascular spaces (87), CSF volume (88), pulsatile CSF flow (89), and brain water metabolism (90). It is useful to investigate the potential effects of specific factors on the CSF flow patterns that cannot be measured. Because symptoms and conclusions are more explicit, computational models are highly valuable. In practice, Bilston et al. (87) simulated the CSF flow in the perivascular space based on fluid mechanics, finding it arterial-driven and that an increased subarachnoid pressure assists the flow toward the central canal. Moreover, the simulation can speculate that the deformation of perivascular spaces will increase the CSF flow rate, which gives a new perspective on the impact of structure deformation in ON CSF flowing spaces. Buishas et al. (91) utilized the Starling forces that govern the passive exchange of water between the capillary microcirculation and the ISF, successfully demonstrating that the production of CSF is periventricular or in the choroid capillaries to the ventricles. Results of both simulations have been proved in later experiments.

## Future direction

Intracranial CSF circulation has been investigated to a considerable extent. However, the corresponding flow in the ON is largely unexplored (29). The narrow and single exit point of the ON subarachnoid space makes the CSF flow here different from that in the brain.

According to Killer's research, the subarachnoid space in the ONs is divided into different cavities by trabeculae and septae (32). However, the connectivity between the subarachnoid space in the ON and that in the cranium is not fully understood. Further studies are warranted to quantify the connectivity between these two cavities and how differences between them would affect the CSF circulation in the ON.

Moreover, the relationship between eye movements and the CSF circulation in the ON deserves further investigation. The functions of eye movements in human REM sleep are still unknown. Eye movements have been hypothesized to drive the CSF flow in the ONs.

## Conclusion

This review described the anatomy and CSF flow in the ON and cranium, as well as research tools for CSF dynamics. The role of the glymphatic system in the brain and the relationship between CSF flow and ophthalmic and neurological diseases were emphasized. Potential mechanisms and driving forces of CSF circulation in the ON are still uncertain. Several questions about ON CSF dynamics remain to be answered. The eye movement was speculated to be the driving force of CSF exchange between the ON and cranial subarachnoid spaces. We are interested in analyzing the pressure gradients in the ON and their impact on the visual pathway.

## Author contributions

XW and JS contributed to the conception of the manuscript and wrote the first draft of the manuscript. QL and TL wrote sections of the manuscript. All authors contributed to manuscript revision, read, and approved the submitted version.

## Funding

This study was funded by the National Natural Science Foundation of China (12002025 and U20A20390) and the 111 Project (B13003).

## Conflict of interest

The authors declare that the research was conducted in the absence of any commercial or financial relationships that could be construed as a potential conflict of interest.

## Publisher's note

All claims expressed in this article are solely those of the authors and do not necessarily represent those of their affiliated organizations, or those of the publisher, the editors and the reviewers. Any product that may be evaluated in this article, or claim that may be made by its manufacturer, is not guaranteed or endorsed by the publisher.
